# Microcystic/Reticular Schwannoma of the Skin: A Report of a Rare Case and Brief Literature Review

**DOI:** 10.7759/cureus.80343

**Published:** 2025-03-10

**Authors:** Vidya Medepalli, Athena Cohen, András Schaffer, Eric Delgado Rendon, Marc Inglese

**Affiliations:** 1 Dermatology, University of Central Florida, Tallahassee, USA; 2 Dermatology, Dermatology Associates of Tallahassee, Tallahassee, USA; 3 Dermatology, Tulane University School of Medicine, New Orleans, USA; 4 Dermatology, Florida State University College of Medicine, Tallahassee, USA

**Keywords:** benign peripheral nerve sheath tumor, microcystic schwannoma, reticular morphology, reticular schwannoma, skin

## Abstract

Schwannomas, categorized under benign peripheral nerve sheath tumors, exhibit a range of rare histological variants, including ancient, plexiform, pseudoglandular, epithelioid, and microcystic/reticular (MR). These variants pose a significant diagnostic challenge. In this report, we describe a case of a 34-year-old female who presented with a two-year history of a mass on her upper back. Histopathological examination revealed a well-defined and encapsulated subcutaneous nodule displaying a biphasic architecture. The predominant component consisted of hypocellular spindle cells forming reticular and cystic spaces adjacent to compressed hypercellular areas. Immunohistochemistry displayed diffuse and strong positivity for S-100 and SOX-10 while lacking expression of specific melanocytic markers, anaplastic lymphoma kinase (ALK), and cytokeratins (CKs). Intracapsular spindle cells were positive for epithelial membrane antigen (EMA). Based on these distinctive features, we arrived at the diagnosis of an MR schwannoma, an entity rarely reported in the skin. We will discuss the differential diagnosis of cutaneous neoplasms exhibiting reticular and microcystic patterns.

## Introduction

Schwannomas are a type of benign peripheral nerve sheath tumor. The majority of cases occur sporadically in young to middle-aged adults as isolated lesions in various organs, including the skin [1-4]. A minority of cases have syndromic associations, including neurofibromatosis type 2-related schwannomatosis, SMARCB1/LZTR1-related-schwannomatosis, and 22q-related schwannomatosis [2-4]. Histologically, classical schwannomas have an epithelial membrane antigen (EMA)-positive perineural capsule, biphasic areas of varying cellularity of spindle cells (Antoni A and B), nuclear palisading (Verocay bodies), and hyalinized vessels [2,3]. Immunohistochemically, they display diffuse S-100 protein and SOX-10 positivity [2].

Rare histological variants of schwannoma, including epithelioid, microcystic/reticular (MR), glandular, pseudoglandular, neuroblastoma-like, and plexiform schwannoma, have been identified in various organs, including the skin [2,3,5-9]. The first cases of MR schwannoma were initially described by Liegl et al. in 2008 [10]. This variant is generally found in visceral locations, often in the gastrointestinal tract, but recent reports have shown that it may also have a predilection for the skin [11]. There have been 47 MR schwannoma cases reported in the literature, with 16 of those cases arising in cutaneous tissue [10-15]. Herein, we report a case of a cutaneous MR schwannoma to aid in the diagnosis of this rare tumor.

## Case presentation

**Figure 1:**
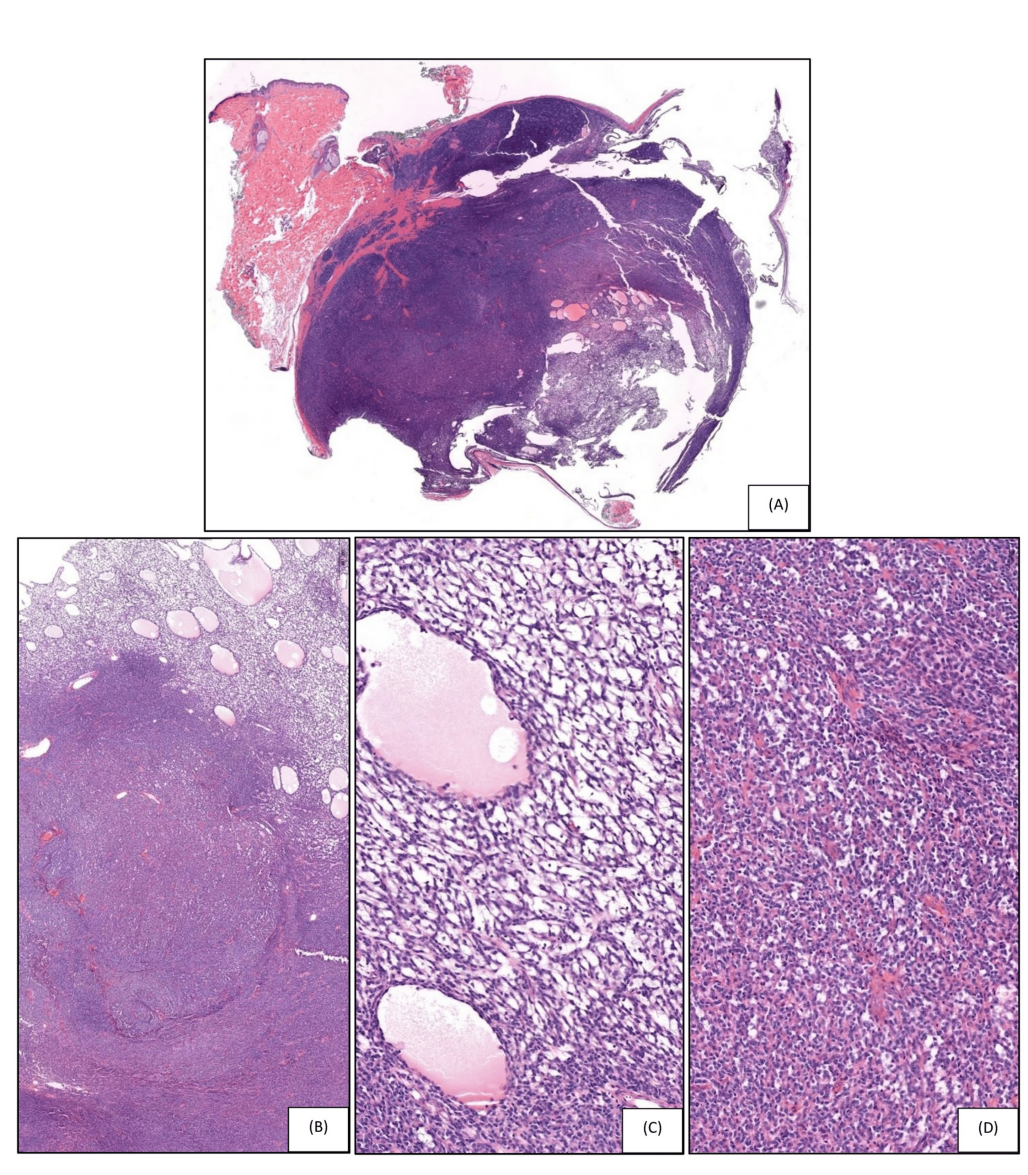
Microscopic examination of the excision specimen reveals a well-defined, encapsulated subcutaneous nodule (A) (hematoxylin and eosin (H&E), ×0.8). H&E staining demonstrates loose and tight reticular areas (B) (H&E, ×2.7), loose reticular areas with microcysts containing serum (C) (H&E, ×18.4), and tight reticular areas of the neoplasm (D) (H&E, ×18.4).

**Figure 2:**
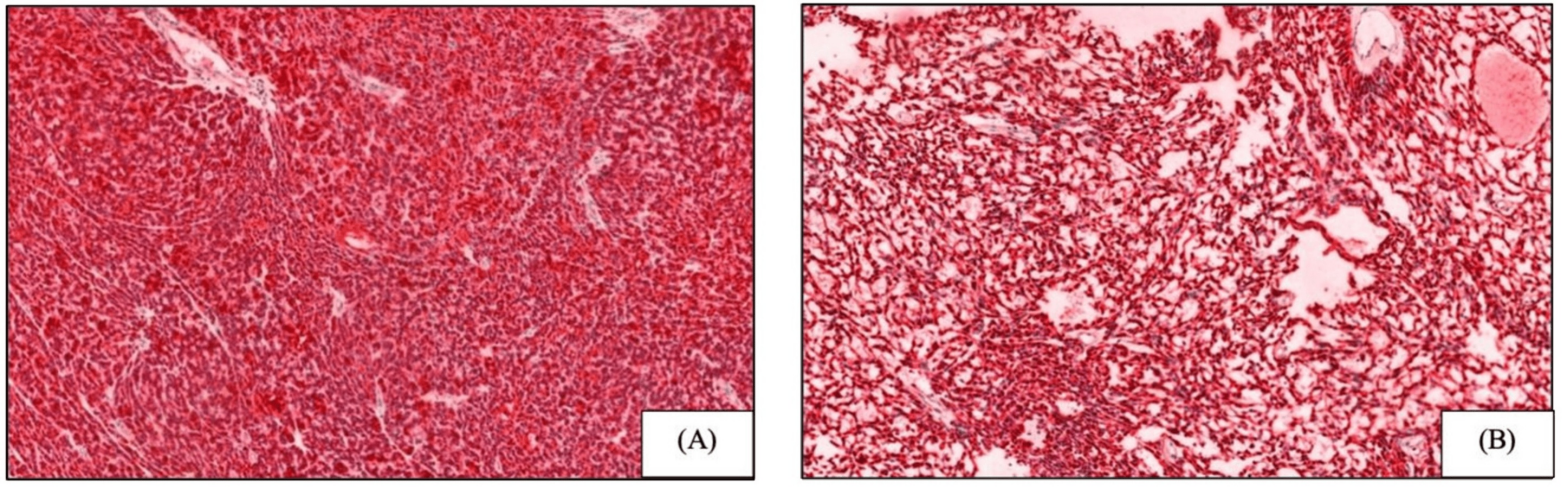
Immunohistochemical staining for S-100 shows diffuse positivity in the tightly packed areas (A) (S-100, ×10) and the loose reticular areas (B) of the neoplasm (S-100, ×10).

**Figure 3:**
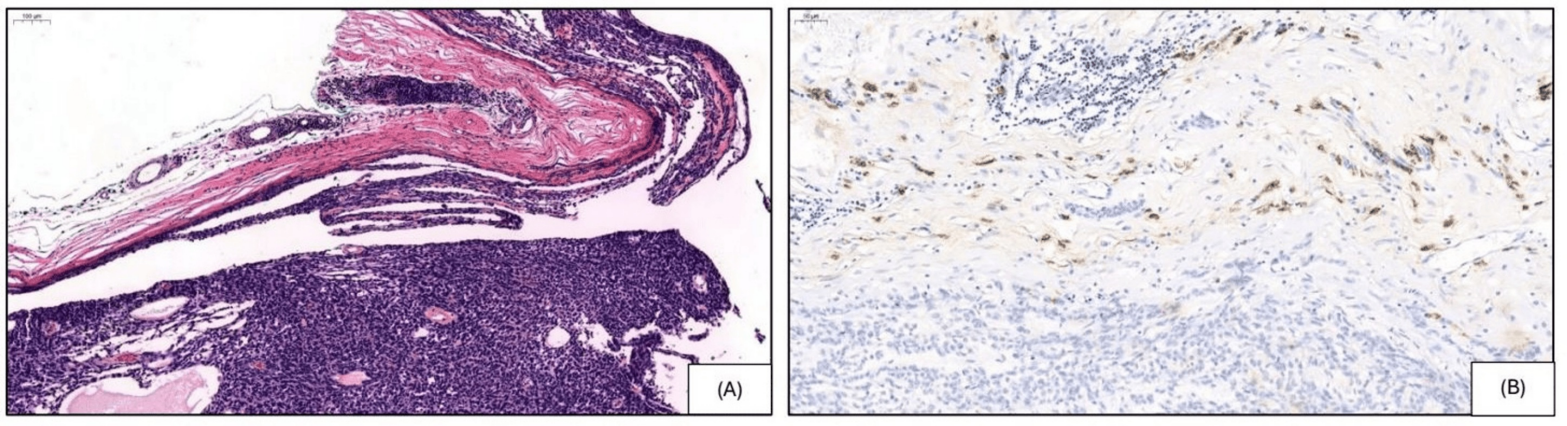
Hematoxylin and eosin (H&E) staining shows the fibrous capsule (A) (H&E, ×9.2) with epithelial membrane antigen (EMA) positivity (B) (EMA, ×16.2).

A 34-year-old female presented with an approximately 1.5 x 1.5 cm skin colored nodule on the right superior medial back. The clinical impression was an epidermal inclusion cyst. The patient had no family history of neurofibromatosis. Upon excision of the lesion, no cyst or cavity was identified by gross examination. Microscopically, the lesion revealed an encapsulated hypo- and hypercellular nodule within the subcutis (Figures 1A-1D). The hypocellular area exhibited spindle cells in a net-like reticular growth pattern with cystic spaces filled with eosinophilic fluid. The hypercellular area is composed of bland ovoid cells forming narrow slit-like reticular spaces. Focally, tumor cells formed small, irregular, slit-like spaces and basement membrane-like deposits. The cells displayed hyperchromasia, prominent nucleoli, and scant eosinophilic cytoplasm. The stroma showed focal scant sclerosis, and hyalinized vessels were not seen. There were only occasional mitotic figures (~1 mitosis per high-power field (HPF)) present. Immunohistochemically, the lesional cells were diffusely positive for S-100 and SOX-10 in the tightly packed (Figure 2A) and reticular areas of the neoplasm (Figure 2B). Type IV collagen was negative on the solid areas but positive on the reticular regions of the neoplasm. EMA showed weak positivity within the fibrous capsule (Figures 3A-3B). Lesional cells were negative for p63, BerEp4, Melan-A, human melanoma black 45 (HMB-45), preferentially expressed antigen of melanoma (PRAME), neuron-specific enolase (NSE), and chromogranin. Cytokeratins (CKs) including AE1/AE3, CK5, and CK20 were also negative. INI1 (SMARCB1) expression was retained in the tumor. Ki-67 staining marked less than 10% of tumor cells. Based on the morphological and immunohistochemical features, the diagnosis of MR schwannoma was made.

## Discussion

The MR variant of schwannoma poses significant diagnostic challenges due to its rarity, unclear clinicopathological characteristics, and shared features with malignant neoplasms. Table 1 describes the clinicopathological features of dermal and subcutaneous MR schwannomas published in the literature as of December 2024. Our case illustrates one of the presentations of MR schwannoma in the skin. In the review of the literature, the average patient age for MR schwannoma occurrence is 40.5 years old, with no gender predilection. The majority of MR schwannoma cases presented as solitary nodules and were found on the upper extremity and trunk with lesser incidence on the lower extremity and head and neck (Table 1). The demographics and clinical presentation of our case were similar to previously published cases, but uniquely presented in the skin.

**Table 1:** Summary of the clinicopathologic features of cutaneous reticular schwannomas published as of December 2024, including the present case. F: Female; M: Male

Case number: reference	Sex	Age	Site	Location	Size (cm)	Growth pattern
1: Liegl et al. [10]	F	50	Arm	Subcutis	2	Circumscribed, encapsulated
2: Liegl et al. [10]	F	56	Back	Subcutis	0.5	Circumscribed, encapsulated
3: Lisle et al. [7]	F	34	Finger	Dermis	NA	Circumscribed, encapsulated
4: Luzar et al. [15]	M	28	Back	Dermis	0.5	Circumscribed, partially encapsulated
5: Luzar et al. [15]	F	30	Upper arm	Dermis	0.7	Circumscribed, partially encapsulated
6: Luzar et al. [15]	M	55	Upper arm	Dermis	1.0	Circumscribed, partially encapsulated
7: Georgescu et al. [14]	M	43	Retroauricular area	Subcutis	2.0	Circumscribed, unencapsulated
8: Bedir et al. [12]	M	28	Pilonidal sinus	Subcutis	2.0	Not reported
9-16: Fritchie et al. [11]	Unreported	Unreported	Unreported	Dermis	Unreported	Mixed circumscription, three out of eight cases were encapsulated
17: Present case	F	34	Back	Subcutis	2	Circumscribed, encapsulated

Histologically, classic schwannomas usually have characteristic Antoni A areas, Antoni B areas, Verocay bodies, perivascular hyalinization, and an EMA-positive perineural capsule [2,3]. MR schwannomas are well-circumscribed but may lack a fibrous capsule [3,13]. MR schwannomas usually lack organization into Antoni A and Antoni B areas and rarely have perivascular hyalinization [12,14]. These neoplasms possess a unique reticular and cystic architecture with bland spindle-shaped Schwann cells with eosinophilic cytoplasm among a myxoid matrix [3,12,14]. Similar to other variants, mitotic activity is generally absent or very low [16].

Our case exhibited features of classic and MR schwannomas, having hypercellular areas of solid sheets of lesional cells, hypocellular areas of spindle cells forming reticular and cystic spaces, and low mitotic activity. Similarly to all schwannoma variants, our case had diffuse S-100 positivity and stained negative for CKs and melanocytic markers [3]. Our case showed a biphasic staining pattern for type IV collagen, with expression only in the reticular areas of the neoplasm. In one case of MR schwannoma, type IV collagen was expressed in the cells lining the cystic spaces [7].

The differential diagnosis of MR schwannomas is challenging and requires an extensive immunohistochemical characterization (Table 2) [7,10,15,17-35]. Benign neoplasms on the differential include reticular perineurioma, nerve sheath myxoma, myxoid variant of cellular neurothekeoma, cutaneous lipomatous neurofibroma, cutaneous myoepithelioma, and sebaceoma. MR schwannomas should also be distinguished from macrocystic schwannomas, which are characterized by a large, fluid-filled cyst resulting from tumor degeneration. The malignant lesions for differential diagnostic consideration for our case include extraskeletal myxoid chondrosarcoma (EMC), primary cutaneous mucinous carcinoma, myxoid malignant peripheral nerve sheath tumor, myxoid variant of dermatofibrosarcoma protuberans, adenoid cystic basal cell carcinoma, and cribriform apocrine carcinoma. S-100 immunohistochemistry is critical for the diagnostic workup. A lack of diffuse S-100 positivity rules out myxoid neurothekeoma, reticular perineurioma, sebaceoma, and myxoid variant of dermatofibrosarcoma protuberans. Nerve sheath myxomas can resemble cutaneous MR schwannoma as they are well-circumscribed tumors in the dermal or subcutaneous tissue with epithelioid or spindled Schwann cells with diffuse S-100 positivity. It is composed of spindle cells arranged in a reticular pattern among a myxoid background [14]. The nested architecture of nerve sheath myxomas and their lack of MR growth help differentiate them from MR schwannoma [14,15]. Immunohistochemical differences between MR schwannoma and the other neoplasms on the differential further distinguish the lesion (Table 2). There is a stark difference in prognosis and treatment between MR schwannoma compared to malignant neoplasms. Therefore, it is critical for clinicians to accurately diagnose these schwannoma variants to avoid unnecessary surgery and distress for patients. However, the limited data outlining the diagnostic criteria and clinicopathological characteristics of MR schwannoma make this difficult. Increased understanding and better recognition of an MR schwannoma are essential to prevent diagnostic pitfalls.

**Table 2:** Pathological differential diagnosis of reticular schwannoma. IHC: Immunohistochemistry; EMA: Epithelial membrane antigen; SMA: Alpha-smooth muscle actin; GFAP: Glial fibrillary acidic protein; NSE: Neuron-specific enolase; NGFR: Nerve growth factor receptor; CK: Cytokeratin; Ber-EP4: Antihuman epithelial antigen; BCL-2: B-cell lymphoma 2; GCDFP: Gross cystic disease fluid protein; CEA: Carcinoembryonic antigen

Differential diagnosis	Typical age group	IHC	Circumscription	Presumed cell of origin	Recurrence rate	Reference
Benign						
Reticular perineuroma	Fourth and fifth decade	Positive stains: EMA, Claudin-1 and Glut-1; Negative stains; S100, SMA, and GFAP negative	No	Perineural cells	5%	Schaefer et al. [30]
Nerve sheath myxoma (neurothekeoma)	Fourth decade	Positive stains: S100 (diffuse), GFAP, CD57, Collagen IV, EMA, NSE; Negative stains: CD10, HMB45, CD63 (NKI/C3), SMA, Neurofilament	Well-circumscribed	Peripheral nerve sheath	47%	Fetsch et al. [21]
Myxoid variant of cellular neurothekeoma	First three decades	Positive stains: NKI/C3 and Ki-M1p; Negative stains: S100 protein or NGFR	No	Unknown	High recurrence rate with local excision	Chen et al. [19], Yun et al. [35]
Cutaneous lipomatous neurofibroma	Unknown	Positive stains: S100, CD34 and vimentin; Negative stains: SMA, desmin	Well-circumscribed	Peripheral nerve sheath	Unknown	Val-Bernal et al. [34]
Cutaneous myoepithelioma	Children and adults	Positive stains: S-100 protein, Variable stains: cytokeratin, GFAP, and EMA	Well-circumscribed	Myoepithelial cells	38%	Hornick et al. [24]
Sebaceoma (rippled pattern)	Middle-aged adults	Positive stains: EMA, Ber-EP4, androgen receptor; Negative stains: S100, Melan-A	Yes	Sebaceous cells	11-30%	Yun et al. [35]
Malignant						
Extraskeletal myxoid chondrosarcoma	Fourth to seventh decade	Positive stains: S100, NSE, synaptophysin; Negative: Cytokeratins	No	Uncertain	48%	Kindblom et al. [25]
Primary cutaneous mucinous carcinoma	Sixth decade	Positive stains: CK7, p63, cytokeratin, EMA, GCDFP-15	No	Sweat glands	7% with Mohs surgery	Tillit et al. [33]
Myxoid malignant peripheral nerve sheath tumor	First to fifth decade	Positive stains: S100, SOX10 (variable), Nestin; Negative stains: neurofibromin H3K27me3 (high grade tumors), INI1 (epithelioid variant)	No	Peripheral nerve sheath	27%	Goertz et al. [22]
Myxoid variant of dermatofibrosarcoma protuberans	Third to fifth decade	Positive stains: CD34; Negative stains: S100, SMA, desmin, EMA, factor XIIIa, CD99	No	Fibroblasts	18%	Kutzner et al. [26], Reimann et al. [29], Betti et al. [17], Orlandi et al. [28]
Adenoid cystic basal cell carcinoma	Older adults (>50 years)	Positive stains: Ber-EP4, BCL-2, CK5/6; Negative stains: EMA, CEA, CK20	Yes	Basal keratinocytes	Low	Seretis et al. [31]
Cribriform apocrine carcinoma	Older adults	Positive stains: CK7, GCDFP-15, S100, EMA; Negative stains: SMA, Desmin	No	Apocrine gland	0%	Boettler et al. [18]

## Conclusions

In summary, we describe a rare case of a cutaneous MR schwannoma. With this unique case, we aim to increase clinical and diagnostic awareness of this rare schwannoma variant. Since this variant exhibits overlapping features with other benign and malignant cutaneous neoplasms, broadening the differential diagnosis and extensive immunohistochemical characterization is critical for its accurate diagnosis.
